# Investigate the application of postoperative ctDNA-based molecular residual disease detection in monitoring tumor recurrence in patients with non-small cell lung cancer——A retrospective study of ctDNA

**DOI:** 10.3389/fonc.2023.1098128

**Published:** 2023-04-06

**Authors:** Xuefei Zhang, Youguo Zhang, Shanli Zhang, Sha Wang, Peng Yang, Changhong Liu

**Affiliations:** ^1^ Department of Thoracic Surgery, The Second Hospital of Dalian Medical University, Dalian, Liaoning, China; ^2^ Geneseeq Research Institute, Nanjing Geneseeq Technology Inc., Nanjing, Jiangsu, China

**Keywords:** NSCLC1, ctDNA2, molecular residual disease (MRD)3, postoperative 4, recurrence risk 5

## Abstract

**Purpose:**

To evaluate whether postoperative circulating tumor DNA (ctDNA) in plasma of patients with non-small cell lung cancer (NSCLC) can be used as a biomarker for early detection of molecular residual disease (MRD) and prediction of postoperative recurrence.

**Methods:**

This study subjects were evaluated patients with surgical resected non-small cell lung cancer. All eligible patients underwent radical surgery operation followed by adjuvant therapy. Tumor tissue samples collected during operation were used to detect tumor mutation genes, and blood samples collected from peripheral veins after operation were used to collect ctDNA. Molecular residue disease (MRD) positive was defined as at least 1 true shared mutation identified in both the tumor sample and a plasma sample from the same patient was.

**Results:**

Positive postoperatively ctDNA was associated with lower recurrence-free survival (RFS).The presence of MRD was a strong predictor of disease recurrence. The relative contribution of ctDNA-based MRD to the prediction of RFS is higher than all other clinicopathological variables, even higher than traditional TNM staging. In addition, MRD-positive patients who received adjuvant therapy had improved RFS compared to those who did not, the RFS of MRD-negative patients receiving adjuvant therapy was lower than that of patients not receiving adjuvant therapy.

**Conclusions:**

Post-operative ctDNA analysis is an effective method for recurrence risk stratification of NSCLC, which is beneficial to the management of patients with NSCLC.

## 1 Introduction

Lung cancer is one of the most common malignancies in the world. According to the epidemiological statistics, lung cancer has ranked sixth among the top 20 major causes of death in the world, and is the malignant tumor with the highest morbidity and mortality among males (1–3). Only about 25% of all patients with non-small cell lung cancer (NSCLC) survived more than five years. The 5-year survival rate for patients with NSCLC was about 25%. The ratio was decreased to less than 6% for metastatic NSCLC even after cytotoxic chemotherapy (4). Up to now, the prognosis of NSCLC is mainly evaluated based on clinicopathological parameters such as TNM stage, pathological subtype and tumor differentiation degree (5). The 2020 edition of the National Comprehensive Cancer Network Guidelines (NCCN) recommends adjuvant chemotherapy after radical surgery for the patients with stage IB-IIIA NSCLC who have high risk for recurrence (6).

However, a significant proportion of patients with low risk of recurrence based on these prognostic indicators experienced tumor recurrence, while some patients with high risk of recurrence still remained recurrence-free after long-time follow-up. At the same time, the outcome of some patients with high recurrence risk was not improved by adjuvant therapy (7). Clinicians urgently need a more effective method to identify patients at high risk of tumor recurrence and who are likely to benefit from adjuvant therapy.

In recent years, liquid biopsy has been introduced into clinical practice as a valuable tool for early diagnosis of recurrence and metastasis of non-small cell lung cancer (NSCLC) (8–12). Circulating tumor DNA (ctDNA) mainly comes from the small fragments of DNA released by apoptotic or necrotic tumor cells in tumor tissues into the blood system and the DNA released by the lysis of circulating tumor cells or micrometastases in the blood (13). Plasma ctDNA analysis can increase the chance of identifying targeted mutations, and is considered as an alternative to tissue biopsy. It has been widely used for early diagnosis, prognostic stratification, disease monitoring and treatment response assessment in different cancer types (14–16). Liquid biopsy techniques based on plasma ctDNA analysis can monitor and track personalized disease-related markers at the molecular level, and can be used to detect molecular residual desease (MRD) after treatment (10, 17, 18). In the TRACERx study, Addosh et al (19) found that ctDNA test after surgery could detect tumor recurrence or metastasis earlier than imaging. Recent trials using personalized mutation detection or CAPP-seq have also demonstrated the potential of ctDNA for MRD detection and early recurrence prediction, especially in post-surgical patients awaiting adjuvant therapy (20, 21). These studies were helpful to evaluate the prognostic value of post-operative ctDNA status in NSCLC. In addition, previous studies mainly focused on detecting MRD after the completion of adjuvant therapy. Few reports have discussed about whether the MRD detection based on the definition of post-operative ctDNA status could guide post-operative adjuvant therapy for NSCLC patients. Also, the utility of dynamic changes in serial ctDNA to predict the risk of relapse during disease surveillance has not been well characterized.

This study collected tumor pathological diagnosis information, post-operative plasma ctDNA detection results, post-operative adjuvant therapy and recurrence status of 73 patients with NSCLC who received radical surgery. The role of MRD detection based on post-operative plasma ctDNA in the prediction of recurrence and metastasis was retrospectively analyzed. It is expected that this study could provide some guidance on personalized treatment according to the molecular characteristics of NSCLC patients.

## 2 Materials and methods

### 2.1 Patients and samples

We enrolled 108 stage I–III NSCLC patients to evaluate the clinical utility of serial ctDNA monitoring from May 2016 to October 2021. Thirty-five patients were excluded due to pathologic diagnosis of stage IV or double/multiple primary lung cancer, the remaining 73 patients were included in the study. According to the latest version of the National Comprehensive Cancer Network (NCCN) guidelines and the patient’s pathologic diagnosis, all patients eligible for postoperative adjuvant therapy are recommended for postoperative adjuvant therapy: (1) For absolutely resected level II to III NSCLC, 4 cycles of adjuvant chemotherapy after operation are recommended; (2) Adjuvant chemotherapy is suggested for sufferers in level IB with high-risk factor after operation. Chest computed tomography (CT) examination was performed at the first, third, sixth, and twelfth months of the first year after discharge, and at least once a year thereafter.

The primary cancer tissues of 73 patients obtained during the operation were pathologically diagnosed in the Department of Pathology, the Second Hospital of Dalian Medical University. 10 ml of whole blood was collected for separation plasma samples in these patients after radical lung cancer surgery and adjuvant therapy. All patients eligible for postoperative adjuvant therapy are recommended for postoperative adjuvant therapy. Chest computed tomography (CT) examination was performed at the first, third, sixth, and twelfth months of the first year after discharge, and at least once a year thereafter. Patients with multiple primary foci, prior malignancy, and incomplete clinicopathological data were excluded from the study. One patient was lost to follow-up, so they were not included in monitoring the efficacy of postoperative adjuvant therapy. The study was approved by the Ethics Review Committee of the Second Hospital of Dalian Medical University, and each patient signed an informed consent.

### 2.2 DNA extraction and targeted next generation sequencing

Genomic DNA from FFPE samples and white blood cells were extracted using the QIAamp DNA FFPE Tissue Kit (Qiagen). Plasma was extracted from about 10 ml whole blood in EDTA coated tubes within 2 h of blood withdrawing, and circulating cell free DNA (cfDNA) was extracted using the QIAamp Circulating Nucleic Acid Kit (QIAGEN). Genomic DNA from white blood cells was extracted using DNeasy Blood & Tissue Kit (Qiagen,Germany) and used as normal control. All DNA concentration and purity were qualified by Nanodrop2000 (Thermo Fisher Scientific). All DNA samples were also quantified by Qubit 3.0 using the dsDNA HS Assay Kit (Life Technologies) according to the manufacturer’s protocol.

Sequencing libraries were constructed using KAPA Hyper Prep kit (KAPA Biosystems) with an optimized manufacturer s instructions. In brief, cfDNA or DNA was experienced with endrepairing, A-tailing, adapter ligation and size selection using Agencourt AMPure XP beads (Beckman Coulter). Libraries were then subjected to PCR amplification and purification before targeted enrichment. The size distribution of libraries was measured by Agilent Technologies 2100 Bioanalyzer (Agilent Technologies). The enriched libraries were sequenced on Illumina Hiseq 4000 NGS platforms to cover mean depths of at least 1,000, 3,000, and 100, for FFPE, cfDNA, and blood, respectively (22).

Single nucleotide variants (SNVs) and short insertions/deletions (indels) were identified using VarScan2 2.3.9 (23) with minimum variant allele frequency threshold set at 0.01 and p-value threshold for calling variants set at 0.05 to generate Variant Call Format (VCF) files. All SNVs/indels were annotated with ANNOVAR, and each SNV/indel was manually checked with the Integrative Genomics Viewer (IGV). Copy number variations (CNVs) were identified using ADTEx 1.0.4 (24).

For each patient, 1 true variant detected in the tumor tissue was measured for its presence in the plasma. At least 1 true shared mutation identified in both the tumor sample and a plasma sample from the same patient was defined as positive for ctDNA. ctDNA levels were quantifiedas the fraction of mutant alleles ×100.

### 2.3 Statistical analysis

The primary outcome looked at was recurrence-free survival (RFS), defined as the period between the patient’s surgery operation and the first confirmation of local or distant tumor recurrence, or death from any cause. The data were analyzed by SPSS 25.0 software. The chi-square test was used to assess the association of clinical characteristics and overall recurrence between postoperative ctDNA positive and negative subgroups, and the independent sample T test was used to compare quantitative data. Kaplan-meier analysis was used to investigate the relationship between progression-free survival (PFS) and postoperative plasma ctDNA status, TNM stage, tumor pathological type, degree of differentiation, and adjuvant therapy.

Cox regression analysis of statistically significant variables was used to analyze the correlation between tumor recurrence, progression-free survival (RFS) and postoperative plasma ctDNA status, TNM stage, tumor pathological type, degree of differentiation, and adjuvant therapy. Thus, the application value of postoperative plasma ctDNA status in the evaluation of prognosis of patients with NSCLC was obtained. P value < 0.05 was defined as statistically significant difference.

## 3 Results

### 3.1 Patient characteristics

The clinicopathological features of the enrolled 73 patients were shown in **Table 1**. The mean age of patients at diagnosis was 59 years (range: 47-84 years). Among all the patients, 35.6% were male, 64.4% were female, 95.9% were pathological type of lung adenocarcinoma, 30 patients (41.1%) had disease stage IB or above, and 21 patients (28.8%) had ctDNA detected in postoperative blood samples. Thirty-one patients (42.5%) received adjuvant therapy. All patients were followed for 4 to 67 months, with a median of 29 months. At the last follow-up, 21 patients (28.8%) had relapsed, and 51 patients (69.9%) had not relapsed or metasasized, except one patient who was lost to follow-up. The median pretreatment ctDNA level was 0 (range 0–38.2), and the median ctDNA level ctDNA after chemotherapy was 0 (range 0–31.6).

**Table 1:** Patient clinicopathological characteristics.

Characteristics	All patients (N = 73)
Gender (%)	
Male	26 (35.6)
Female	47 (64.4)
Age (years)	
Median (range)	59 (37-84)
Smoking status (%)	
Yes	14 (19.2)
No	59 (80.8)
Hypertension (%)	
Yes	19 (26)
No	54 (74)
Diabetes (%)	
Yes	10 (13.7)
No	63 (86.3)
Histology (%)	
Adenocarcinoma	70 (95.9)
Squamous cell carcinoma	3 (4.1)
Differentiation (%)	
Microinvasive	5 (6.8)
High	5 (6.8)
Moderately	43 (58.9)
Low	17 (23.3)
Mucous	3 (4.1)
pTMN stage (%)	
IA	43 (58.9)
IB	9 (12.3)
IIA	1 (1.4)
IIB	8 (11.0)
IIIA	11 (15.1)
IIIB	1 (1.4)
T stage (%)	
T1	52 (71.2)
T2	19 (26.0)
T3	1 (1.4)
T4	1 (1.4)
N stage (%)	
N0	53 (72.6)
N1	7 (9.6)
N2	13 (17.8)
ctDNA, pretreatment	
Median (range)	0 (0-38.2)
ctDNA after chemotherapy	
Median (range)	0 (0-31.6)
Adjuvant therapy (%)	
Yes	31 (42.5)
No	41 (56.2)
ctDNA Status,after chemotherapy (%)	
ctDNA Positive	13 (17.8)
ctDNA Negative	56 (76.7)
Undeteced	4 (5.5)
Recurrence (%)	
Yes	21 (28.8)
No	51 (69.9)

### 3.2 Prognostic value of the post-operative ctDNA

**Figure 1:**
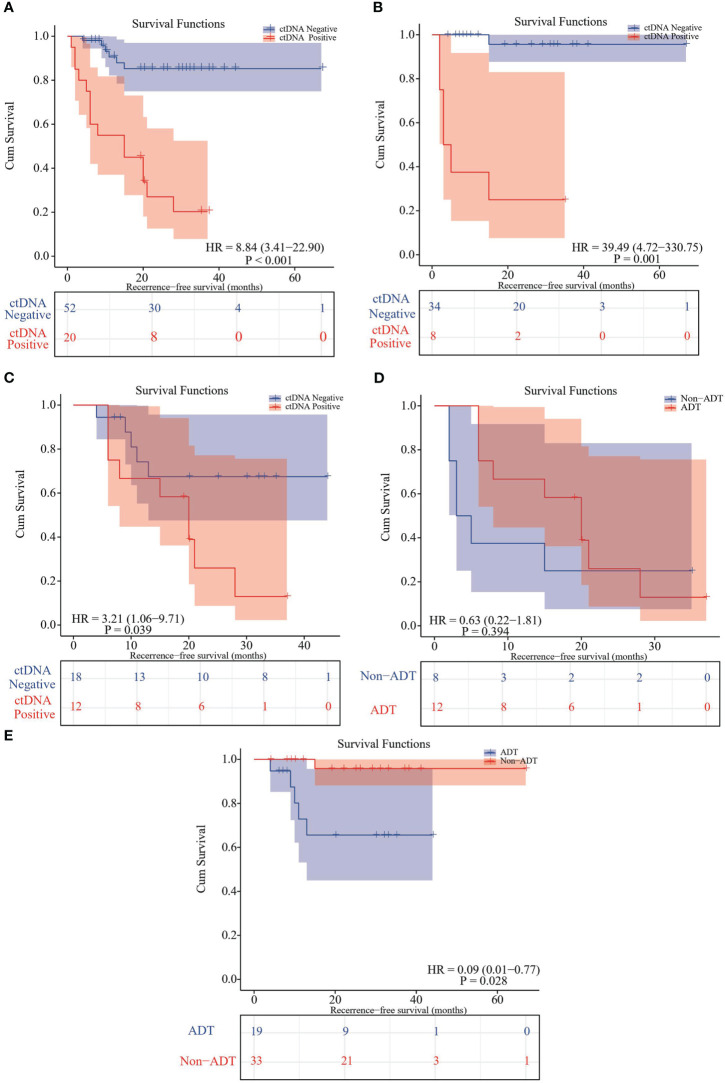
Kaplan–Meier curve in patients stratified by postsurgical ctDNA status. **(A)** Kaplan-Meier curve displaying RFS stratified by ctDNA-based MRD status. **(B)** Kaplan-Meier curve comparing RFS stratified of MRD-positive and MRD-negative subsets in TNM stage IA patients. **(C)** Kaplan-Meier curve comparing RFS stratified of MRD-positive and MRD-negative subsets in TNM stage IB-III patients. **(D)** Kaplan-Meier analysis comparing RFS between MRD-positive patients receiving adjuvant therapies and not receiving adjuvant therapy. **(E)** Kaplan-Meier analysis comparing RFS between MRD-negative patients receiving adjuvant therapies and not receiving adjuvant therapy.

Univariate and multivariate analysis were performed to evaluate the relationship between RFS and clinicopathological variables (**Table 2**). The result showed that only pTNM stage and adjuvant therapy were correlated with RFS (P<0.05). We further evaluated the relationship between postoperative ctDNA status and tumor recurrence/metastasis in NSCLC. Data analysis showed that 71.4% (15 of 21) of patients who were postoperative ctDNA-positive experienced recurrence compared with those who were ctDNA-negative (6 of 52, 11.5%) (**Table 2**). We get the same results as before, post-operative ctDNA-based MRD status is a strong predictor of NSCLC recurrence. The postoperative ctDNA status was significantly correlated with RFS (**Table 2**). The ctDNA-positive patients had significantly worse RFS (HR 8.84, 95% CI 3.41-22.90; P<0.001) (**Figure 1A**). After adjusting for clinicopathological risk factors, postoperative ctDNA status remained an independent risk factor for RFS (HR 7.757, 95%CI 2.787-21.592; P <0.01) (**Table 2**). Survival curves showed that positive MRD was correlated with RFS in both stage IA (HR 39.49, 95%CI 4.72-330.75; P=0.001) (**Figure 1B**)and stage IB-III patients (HR 3.21, 95%CI 1.06-9.71; P=0.039) (**Figure 1C**).

**Table 2:** Recurrence-free survival (RFS) analysis of clinicopathological variables and postoperative ctDNA status in NSCLC patients.

Variables	Univariate analysis			Multivariate analysis		
	HR	95% CI	P value	HR	95% CI	P value
Postoperative ctDNA (n = 72)						
ctDNA status (Positive vs. Negative)	8.765	3.385 - 22.698	< 0.001	7.757	2.787 - 21.592	< 0.001
Age	1.041	0.988 - 1.097	0.131			
Gender (Female vs. Male)	0.764	0.322 - 1.816	0.543			
Smoking status(Yes vs. No)	1.653	0.641 - 4.265	0.299			
Hypertension (Yes vs. No)	0.902	0.330 - 2.463	0.840			
Diabetes (Yes vs. No)	0.930	0.273 - 3.160	0.907			
Histology(Adenocarcinoma vs. Squamous cell carcinoma)	0.698	0.093 - 5.226	0.726			
pTMN stage (IA vs. IB-III)	2.767	1.116 - 6.865	0.023	1.047	0.073 - 14.972	0.973
Adjuvant therapy (Yes vs. No)	2.859	1.151 - 7.100	0.024	1.313	0.092 - 18.775	0.841

In current clinical practice, patients with stage IB-III NSCLC based on clinicopathological factors are usually advised to receive adjuvant therapies after radical surgery, but only a small percentage of patients benefit from adjuvant therapies (6, 25, 26) . In this study, we explored the possibility that ctDNA-based MRD testing could help select eligible NSCLC patients for adjuvant therapy. One patient was excluded in the adjuvant therapy analysis because of loss of follow-up. First, we stratified the patients according to traditional TNM staging. Recurrence was detected in 43 of stage IA patients (16.27%) and of stage IB-III patients (45.16%), respectively. Of the 43 stage IA patients, 3 who received post-operative adjuvant therapy (ADT) did not experience recurrence, while 7 (18%) of those who did not receive ADT. Recurrence was detected in 30 of stage IB-III patients (46.67%) respectively. Of the 30 stage IB-III patients who received ADT (28 patients), 14(50%) experienced tumor recurrence/metastasis, while 2 patients who did not receive ADT remained disease-free (**Table 3**).

**Table 3:** Relationship between postoperative ctDNA-based MRD and tumor recurrence and adjuvant therapy.

MRD			
Tumor recurrence/metastasis	positive	negative	
Yes (%)	15	6	21 (29.2)
No (%)	5	46	51 (70.8)
	20 (27.8)	52 (72.2)	
Adjuvant therapy			
Yes (%)	12	19	31 (43.1)
No (%)	8	33	41 (56.9)
	20 (27.8)	52 (72.2)	

When the effect of ADT on tumor recurrence/metastasis was analyzed in terms of post-operative ctDNA status, 9 (75%) of the 12 patients who were post-operative ctDNA-positive in the stage IB-III patients experienced recurrence. Among the patients with post-operative ctDNA-negative, recurrence was observed in 5 of 19 patients (26.3%) who received ADT (**Table 3**), compared with only 1 of 33 patients who did not receive adjuvant therapy.

For the patients who were post-operative ctDNA-positive, adjuvant therapy could significantly improve RFS (mean RFS: 18.5 months vs. 12.3 months; HR 0.63, 95% CI 0.22-1.81; P=0.394) (**Figure 1D**). In contrast, in the post-operative ctDNA-negative patients, those receiving adjuvant therapy had significantly shorter RFS (mean RFS 32.2 months versus 64.8 months; HR 0.09, 95% CI 0.01-0.77; P=0.028) (**Figure 1E**). However, when stratified the patients according to traditional TNM staging, there was no significant difference in PFS of both stage IA (P=0.48) and IB-III (P=0.21) patients who received ADT and those who did not. After adjustment for other clinicopathological variables, adjuvant therapy bring significant benefit to RFS in the MRD-negative population.

## 4 Discussion

There are correlations between plasma ctDNA and tumor burden, metabolism, proliferative indices, lymphovascular invasion, and the risk of disease recurrence/progression in patients with non-small cell lung cancer (NSCLC) (25). It is possible to use ctDNA profiling to quantify MRD, a disease state associated with medically occult residual disease that can be detected using ctDNA, and inferior disease-free survival (DFS) (26). Plasma ctDNA monitoring has become a new method for prognosis prediction and post-operative monitoring of NSCLC. At present, various methods have been developed for ctDNA detection in solid tumors (3, 21, 27). However, it is difficult to determine the proportion of ctDNA by direct cfDNA quantification. Indirect quantification is based on the sequencing of cfDNA and the abundance of tumor-specific mutations carried in cfDNA (28). One study of 230 lung cancer patients found a positive correlation between postoperative ctDNA and late recurrence rates (27). Based on plasma samples, researchers determined the correlation between ctDNA status and postoperative RFS of patients using ctDNA quantified by highest mutation allele fraction (MAF), ctDNA status has a greater effect on RFS than any one or combination of clinicopathologic risk factors. In this prospective cohort study, we examined 73 NSCLC patients treated with surgical resection with adjuvant therapy to evaluate the utility of ctDNA in disease monitoring and treatment determination.

Several previous studies have shown that positive plasma ctDNA after surgical resection is associated with poor prognosis in lung cancer patients (20, 25, 29, 30). ctDNA serves as a robust biomarker for postsurgical and post-adjuvant chemotherapy risk stratification and early detection of recurrence in NSCLC (31). According to Wu, the prognostic value of MRD detection in patients with NSCLC after definitive surgery was confirmed, especially in those with longitudinal undetectable MRD (32), subgroup analysis suggested that adjuvant therapy may be unnecessary for patients with undetectable MRD. During surgery, ctDNA analysis can detect recurrence and MRD risk stratification of NSCLC. Adjuvant treatment is an independent factor in recurrence-free survival in MRD-positive population (33), Investigators confirmed MRD is a strong predictor for disease relapse, and whether receiving adjuvant therapies is an independent factor for RFS in the MRD-positive population. In this retrospective study, we examined 73 NSCLC patients undergoing radical surgical resection to assess the utility of ctDNA in disease surveillance and treatment determination. In our study, there were several situations: 4 patients who were ctDNA-negative 1-3 days after surgery, but their lessions recurred during subsequent monitoring. Among these 4 patients, one died of acute renal failure in 1 year after surgery, and 2 patients had ctDNA detected in blood tests after recurrence. One patient with brain metastasis did not detect ctDNA at any time point before and after recurrence, or even during the entire post-operative monitoring period. That could be explained by the fact that the blood brain barrier may deter ctDNA from brain lesions releasing into the circulation (31). Another reason for the negative ctDNA test may be the heterogeneity of the primary tumor, as the mutated gene sequence of the recurrent tumor may not be included in the sequencing region of the tumor biopsy and the range of test sequences used in this study.

The majority of patients in our study cohort were in stage I (58.9%). In contrast, most of the previous studies mainly enrolled patients with stage IB and the above stages (26, 34). Our cohort may more closely reflect the real-world distribution of NSCLC patients undergoing radical surgical treatment (32, 35). Currently, tumor recurrence is monitored mainly by imaging techniques. However, some studies have demonstrated the value of longitudinal ctDNA analysis to predict recurrence earlier than imaing (36–38). Our results suggest that post-operative ctDNA-positive is a strong predictor of NSCLC recurrence, and patients with post-operative positive MRD are more likely to relapse than patients with negative MRD. Multivariate Cox analysis also showed that MRD status was an independent factor of RFS. These results suggest that post-operative MRD status can be a powerful indicator of patient prognosis stratification.

In this study, most patients in stage IB and II-III received ADT, while most patients in stage IA did not. Therefore, it is acceptable that adjuvant therapy is associated with shorter RFS across the entire cohort (average ADT PFS 27.3 months vs. average non-ADT PFS 55.7 months). However, in subgroup analysis, two subgroups showed opposite trends: for MRD-positive patients, ADT is favorable to longer RFS, while for MRD-negative patients who received ADT had shorter RFS. The results held true when the analysis was further limited to a subgroup of stage IB-III patients. These results clearly indicate that MRD testing can be a powerful tool in screening patients for ADT.

To sum up, the key findings of this study includes: (1) ctDNA can be used as a biomarker for the prognosis of NSCLC after radical surgery; (2) Early prediction of tumor recurrence can be made by ctDNA-based MRD detection after NSCLC surgery; (3) MRD positive patients will benefit from adjuvant therapy, while adjuvant therapy is not beneficial or even harmful to MRD-negative patients. These findings suggested that ctDNA quantification is a more sensitive monitoring technique than traditional imaging.

There are still some limitations to our study. First, our study is limited by a modest sample size and an absence of validation cohorts. However, the results of our study are consistent with those of others that have shown the potential clinical use of ctDNA analysis as a prognostic predictor. Future studies are still needed to further validate whether ctDNA detection can be adopted in routine clinical care. Secondly, the adoption of negative results needs further verification. Multiple factors may lead to false negative results of ctDNA. Thirdly, Some patients failed to complete the blood test in time for some reasons, resulting in missing genetic test results before and after recurrence.

In conclusion, in this retrospective study, we demonstrate that post-operative ctDNA testing can effectively detect MRD and identify NSCLC patients at high risk of recurrence. Adjuvant therapy based on MRD grouping can achieve greater survival benefit for patients with NSCLC. Our findings reveal the potential clinical use of postoperative ctDNA-based MRD testing in NSCLC patients.

## Data availability statement

The datasets used and/or analyzed during the current study are available from the corresponding author on reasonable request.

## Ethics statement

Written informed consent was obtained from the individual(s) for the publication of any potentially identifiable images or data included in this article.

## Author contributions

CL and XZ conceived the study. CL and XZ provided project management and supervision. XZ, YZ and SZ provided or facilitated the accrual of patient samples, pathology, and/or clinical data. PY and SW performed bioinformatics and genomic analyses. XZ performed statistical analyses. XZ wrote the original draft, with input from all authors. All authors contributed to the article and approved the submitted version.
